# Supplementary material for: Fundamental sex differences in cocaine-induced plasticity of D1R- and D2R-MSNs in the mouse nucleus accumbens core
Source: Biol Sex Differ. 2025 Nov 26;16:102. doi: 10.1186/s13293-025-00785-6 (PMC12659347; doi:10.1186/s13293-025-00785-6)
Supplement: Supplementary file 1 — Supplementary Material 1 [file 13293_2025_785_MOESM1_ESM.docx]

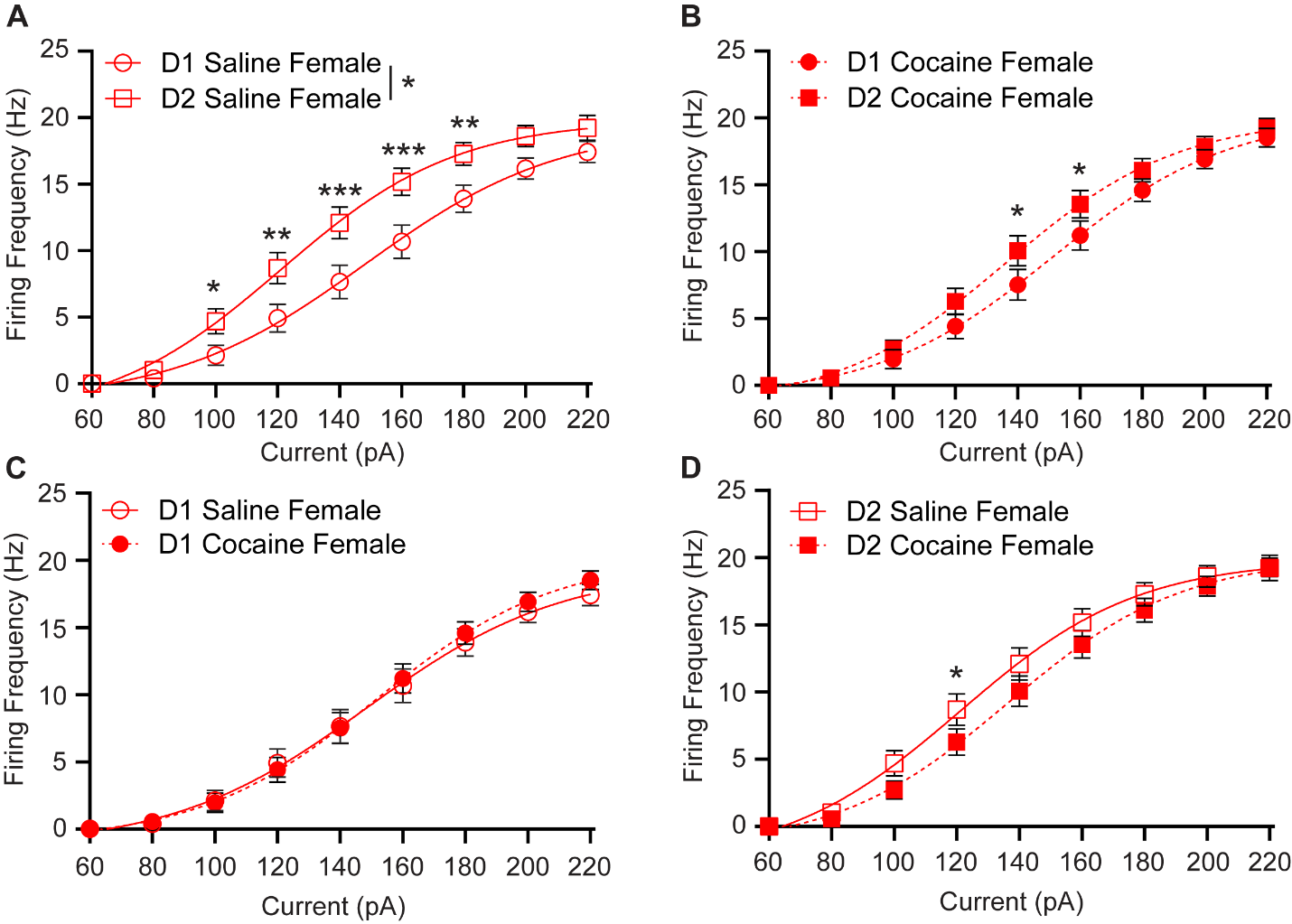


**Supplemental Figure 1:** NAcC neuronal excitability in females collapsed across estrous cycle. **(A)** Summary data for saline-treated D1R-MSN vs D2R-MSN neuronal excitability. **(B)** Summary data for cocaine-treated D1R-MSN vs D2R-MSN neuronal excitability. **(C)** Summary data for cocaine-treated vs saline-treated D1R-MSN neuronal excitability. **(D)** Summary data for cocaine-treated vs saline treated D2R-MSN neuronal excitability. D_1_ receptor; D2R, D_2_ receptor; For main effect comparisons: * *p* < 0.05. For post-hoc comparisons: * *p* < 0.05, ** *p* < 0.01,*** *p* < 0.001. Open circles and squares are from saline treatment, closed circles and squares are from cocaine treatment.
